# Characterizing the Status of Energetic Metabolism of Dinoflagellate Resting Cysts under Mock Conditions of Marine Sediments via Physiological and Transcriptional Measurements

**DOI:** 10.3390/ijms232315033

**Published:** 2022-11-30

**Authors:** Fengting Li, Caixia Yue, Yunyan Deng, Ying Zhong Tang

**Affiliations:** 1 CAS Key Laboratory of Marine Ecology and Environmental Sciences, Institute of Oceanology, Chinese Academy of Sciences, Qingdao 266071, China; 2 Laboratory for Marine Ecology and Environmental Science, Qingdao National Laboratory for Marine Science and Technology, Qingdao 266237, China; 3 Center for Ocean Mega-Science, Chinese Academy of Sciences, Qingdao 266071, China; 4 University of Chinese Academy of Sciences, Beijing 100049, China

**Keywords:** ATP content, energetic metabolism, resting cyst, *Scrippsiella acuminata*, tricarboxylic acid cycle, viability

## Abstract

Similar to the seeds of higher plants, resting cysts, a non-motile, benthic, and dormant stage in the life history of many dinoflagellate species, play vital roles via germination in the seasonal dynamics and particularly the initiation of harmful algal blooms (HABs) of dinoflagellates. It is thus crucial for resting cysts to balance between the energetic catabolism for viability maintenance and the energy preservation for germination during their dormancy. Despite this importance, studies on how resting cysts of dinoflagellates accomplish energetic metabolism in marine sediment have been virtually absent. In this study, using the cosmopolitan HABs-causing species *Scrippsiella acuminata* as a representative, we measured the transcriptional activity of the most efficient pathway of the energy catabolism tricarboxylic acid (TCA) cycle, cell viability (via neutral red staining), and the cellular ATP content of resting cysts under a set of mock conditions in marine sediments (e.g., 4 °C, darkness, and anoxia) for a maximum period of one year. Based on the correlation analyses among the expression levels of genes, cyst viability, and ATP content, we revealed that the TCA cycle was still a crucial pathway of energetic catabolism for resting cysts under aerobic conditions, and its expression was elevated at higher temperatures, light irradiation, and the early stage of dormancy. Under anaerobic conditions, however, the TCA cycle pathway ceased expression in resting cysts, as also supported by ATP measurements. Our results have laid a cornerstone for the comprehensive revelation of the energetic metabolism and biochemical processes of dormancy of resting cysts in marine sediments.

## 1. Introduction

In recent decades, harmful algal blooms (HABs) have occurred frequently around the world and posed a serious threat to coastal ecosystems, fisheries, and human health [1,2,3,4]. Dinoflagellates account for about 75% of HAB events and 40% of all HAB-causing species [5,6]. Dinoflagellates are strong competitors in forming HABs as they possess remarkable adaptive strategies, such as trophic flexibility, toxin production, and allelopathy [3,7,8,9]. The formation of resting cysts is one of these strategies for many dinoflagellates. Dinoflagellates form resting cysts in harsh conditions and are usually associated with gene recombination [10], resistance to adverse environments [10,11], and the initiation and termination of blooms [12,13].

Resting cysts can survive in sediments for more than a century under extreme conditions such as low temperature, darkness, and a lack of oxygen [14,15,16]. The energetic metabolism in resting cysts is very important during dormancy. Due to the atrophy of organelles such as chloroplasts [17], there is no substance synthesis in resting cysts. In recent years, a few studies attempted to explore the energy metabolism pathways within resting cysts [18,19,20]. Deng et al. [18] found that the resting cysts of *Scrippsiella acuminata* (formerly *S. trochoidea*, [21]) showed lower expression levels of the photosynthetic pathway than that in vegetative cells, and the genes involved in the TCA cycle, the glycolytic pathway, and the glyoxalate cycle were still expressed in newly formed cysts under room temperature. Guo et al. [19] reported that genes encoding glucose-6-phosphate isomerase and citrate synthase, two enzymes involved in glycolysis and the TCA cycle, respectively, were up-regulated in *S. acuminata* during the formation of pellicle cysts, but down-regulated in pellicle cysts after formation. According to Wu et al. [20] energy storage and consumption are critical in cyst formation and germination, respectively, and the processing of genetic information is crucial in cyst dormancy. These reports provide a basic understanding of the energetic metabolism in *S. acuminata*.

The most direct source of energy in organisms is adenosine triphosphate (ATP) [22,23], as ATP is the universal energy currency of cells [24]. Therefore, changes in ATP content represent the dynamics of energetic metabolism in resting cysts. The TCA cycle is an energy metabolic pathway in organisms under aerobic conditions and is the hub of carbohydrate, lipid, and protein metabolism [25,26,27]. One molecule of glucose can produce 32 ATP after passing through the TCA cycle [28]. The TCA cycle is a cyclic reaction system consisting of a series of enzymatic reactions in the mitochondrial matrix [29]. The reaction from acetyl coenzyme A (acetyl-CoA) to citrate, catalyzed by citrate synthase (CS), is recognized as the first committed step of the TCA cycle [29,30]. Isocitrate dehydrogenase (IDH) catalyzes the oxidative decarboxylation of isocitrate to α-ketoglutarate and CO_2_, which is considered to be a key regulatory step in the TCA cycle [31,32,33]. Both *CS* and *IDH* genes were used to indicate the effects of fluorine stress on the TCA cycle in *Khitish* rice seedlings [34]. The multienzyme complex of α-ketoglutarate dehydrogenase (α-KGDH) is the key rate-limiting enzyme for the second oxidative decarboxylation reaction in the TCA cycle [35,36]. The *α-KGDH* was used to assess the efficacy of *Withania somnifera* on the TCA cycle in mice [37]. Thus, the overall transcriptional levels of *CS*, *IDH*, and *α-KGDH*, may be adequately used to represent the expression levels of the intracellular TCA cycle pathway.

The armored dinoflagellate *S. acuminata* is a notorious HAB-forming species that has a cosmopolitan distribution covering global oceans (as reviewed in Tang and Gobler, 2012) [38]. Due to its wide geo-distribution, high capability to form cysts both in the laboratory and field, and increasing studies on the species as partly reviewed above, *S. acuminata* has been adopted as a representative species for life history studies on dinoflagellates [18,19,20,39,40,41]. In this study, using *S. acuminata* as a representative dinoflagellate again, we measured the transcriptional activity, cell/cyst viability, and intracellular ATP content of resting cysts under a range of conditions simulated to that possibly observed in the marine sediments. The objective was to gain further insights into the energetic metabolism of resting cysts of dinoflagellates at both the physiological and transcriptional levels, with a focus on the activity of the TCA cycle pathway. Our results lay the foundation for a comprehensive understanding of the molecular mechanisms underlying the energetic metabolism and viability maintenance of resting cysts buried in sediments.

## 2. Results

### 2.1. General Characterization of the Full-Length cDNA Sequences of SaCS (CS in S. acuminata), SaIDH (IDH in S. acuminata), and Saα-KGDH (α-KGDH in S. acuminata)

**Figure 1:**
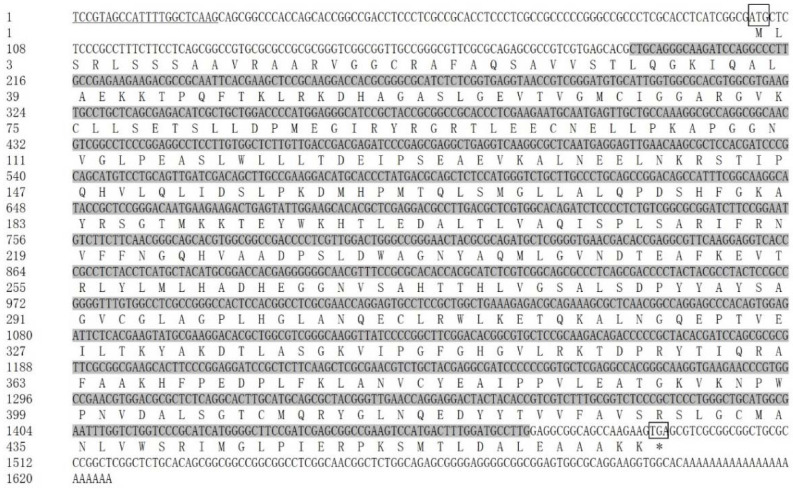
The full-length cDNA sequence and deduced amino acid sequence of *SaCS* (Accession: OP358035). Sequence analysis was performed using NCBI database and numbered on the left; the start and stop codon are in box; stop codon were shown as “*”; the conserved dinoflagellate spliced leader (DinoSL) is underlined in the 5′-UTR; the conserved domain is shaded in dark gray.

The sequencing of *SaCS* yielded 790 bp and 499 bp at the 3′ and 5′ RACE (rapid amplification of cDNA ends) products, respectively. The 1625 bp full-length cDNA sequence of *SaCS* consisted of a 5′-terminal untranslated region (UTR) of 101 bp, a 3′-UTR of 132 bp, and an ORF (open reading frame) of 1392 bp (Figure 1). The ORF had 64.08% GC content and encoded a protein of 463 amino acid residues with a predicted molecular weight of 50.50 kDa and a theoretical isoelectric point of 8.44. The obtained sequence was deposited in GenBank with the accession number (OP358034). From the GenBank database, the dinoflagellates *Karlodinium veneficum* (ADV91159), *Polarella glacialis* (CAE8611289), and *Symbiodinium microadriaticum* (CAE7173768) containing the *CS* sequence were downloaded. The *SaCS* protein shared 68.4%, 82.3%, and 78.3% similarity with the above three species, respectively (Supplementary Materials S1).

**Figure 2:**
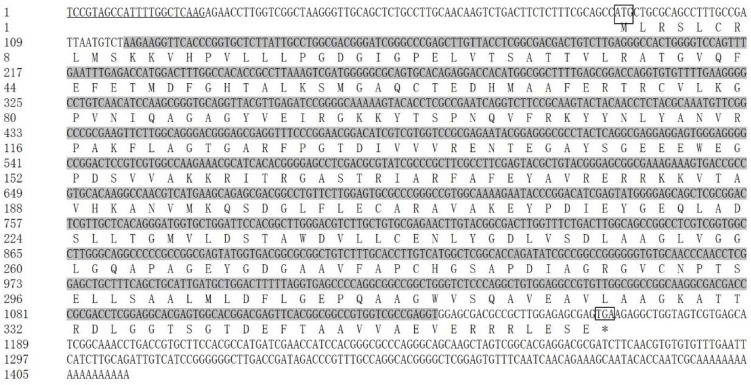
The full-length cDNA sequence and deduced amino acid sequence of *SaIDH* (Accession: OP358035). Sequence analysis was performed using NCBI database and numbered on the left; the start codon ATG and stop codon TGA were boxed; “*” stood for uncoded amino acid; the Dino-SL was underlined in the 5′-UTR; the conserved domain was shaded in dark gray.

For the gene *SaIDH*, the sequencing yielded 791 bp and 537 bp at the 3′ and 5′ RACE products, respectively. The 1413 bp full-length cDNA sequence of *SaIDH* consisted of a 5′-UTR of 86 bp, a 3′-UTR of 247 bp, and an ORF of 1080 bp (Figure 2). The ORF had 62.41% GC content and encoded a protein of 359 amino acid residues with a predicted molecular weight of 38.46 kDa and a theoretical isoelectric point of 5.58. The obtained sequence was deposited in GenBank with the accession number (OP358035). From the GenBank database, the dinoflagellates *S. pilosum* (CAE7159390), *S. necroappetens* (CAE7741129), and *S. natans* (CAE7614210) containing the *IDH* sequence were downloaded. The *SaIDH* protein shared 61.3%, 42.9%, and 57.6% similarity with the above three species, respectively (Supplementary Materials S1).

**Figure 3:**
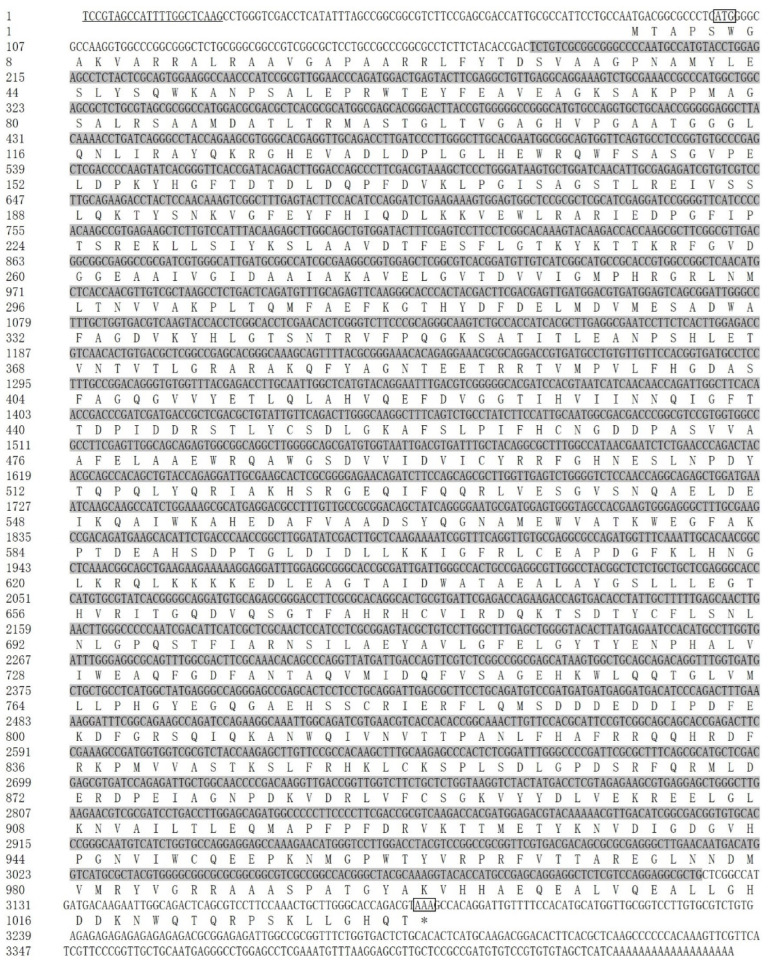
The full-length cDNA sequence and deduced amino acid sequence of *Saα-KGDH* (Accession: OP358036). Sequence analysis was performed using NCBI database and numbered on the left; the start codon ATG and stop codon TGA were boxed; “∗” stood for uncoded amino acid; the Dino-SL was underlined in the 5′-UTR; the conserved domain was shaded in dark gray.

The detected sequence of gene *Saα-KGDH* yielded 1650 bp and 618 bp at the 3′ and 5′ RACE products, respectively. The 3451 bp full-length cDNA sequence of *Saα-KGDH* consisted of a UTR of 85 bp, a 3′-UTR of 264 bp, and an ORF of 3102 bp (Figure 3). The ORF had 58.63% GC content and encoded a protein of 1033 amino acid residues with a predicted molecular weight of 115.05 kDa and a theoretical isoelectric point of 5.94. The obtained sequence was deposited in GenBank with the accession number (OP358036). From the GenBank database, the dinoflagellates *P. glacialis* (CAE8618239), *S. microadriaticum* (OLQ12443), and *S. natans* (CAE7501016) containing the *α-KGDH* sequence were downloaded. The *Saα-KGDH* protein shared 68.3%, 65.4%, and 71.2% similarity with the above three species, respectively (Supplementary Materials S1).

### 2.2. Transcriptional Responses of the Three Targeted Genes in Vegetative Cells

#### 2.2.1. Transcriptional Responses of the Three Targeted Genes at Different Growth Stages

**Figure 4:**
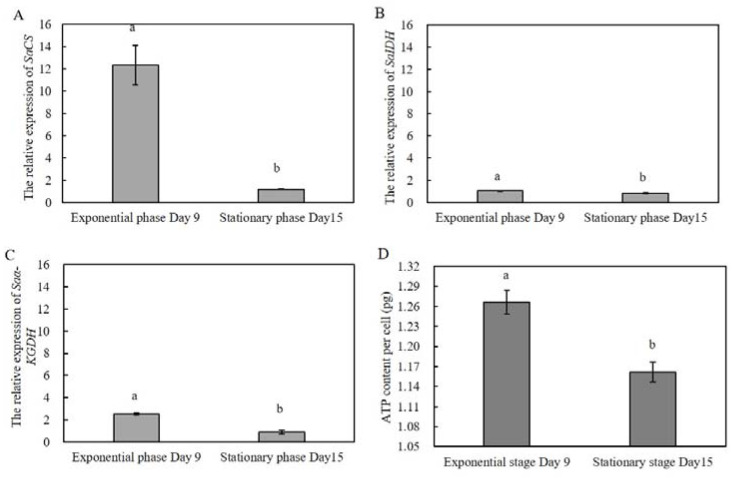
Quantitative expression of *SaCS*, *SaIDH*, *Saα-KGDH*, and ATP content in vegetative cells at different growth cycles. (**A**) stood for *SaCS*; (**B**) stood for *SaIDH*; (**C**)stood for *Saα-KGDH*; (**D**) ATP content. Data were presented in graphs as mean ± standard deviation (SD), subjected to one-way analysis of variance (ANOVA) and a subsequent Tukey’s honestly significant difference test, *n* = 2, *p* < 0.05. “a, b” mean that there were differences between the groups they represent.

The expression levels of *SaCS*, *SaIDH*, and *Saα-KGDH* in the stationary phase were all significantly lower than that in the exponential phase (90.5%, 54.2%, and 62.4% of that at the exponential phase, respectively; Figure 4A–C). The ATP content of vegetative cells at the exponential phase was measured to be significantly higher than that at the stationary phase (Figure 4D), a trend consistent with the expression levels of the three genes regulating the TCA cycle.

#### 2.2.2. Transcriptional Responses of the Three Targeted Genes at Different Circadian Periods

**Figure 5:**
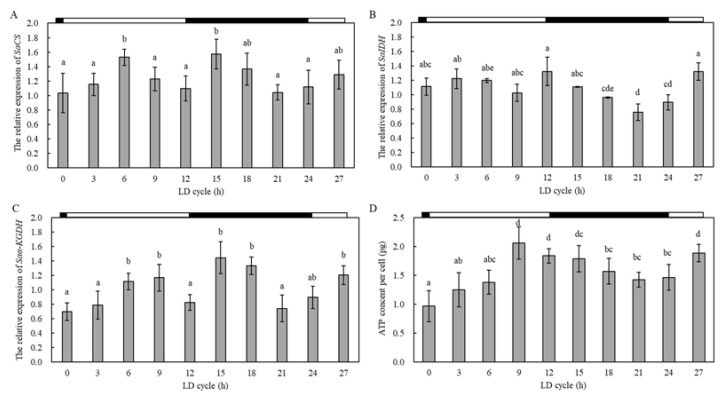
Quantitative expression of *SaCS*, *SaIDH*, *Saα-KGDH*, and ATP content in vegetative cells at different circadian rhythms. (**A**) stood for *SaCS*; (**B**) stood for *SaIDH*; (**C**) stood for *Saα-KGDH*; (**D**) ATP content. The white and black bars at the top of the chart indicated photoperiod and dark period, respectively. Data were presented in graphs as mean ± standard deviation (SD), subjected to one-way analysis of variance (ANOVA) and a subsequent Tukey’s honestly significant difference test, *n* = 10, *p* < 0.05. “a–d” mean that there were differences between the groups they represent, respectively.

The expressions of *SaCS*, *SaIDH*, and *Saα-KGDH* in the vegetative cells exhibited different changing patterns at different circadian periods (Figure 5). For *SaCS* and *Saα-KGDH*, their expression levels increased (0–6 h and 0–9 h, respectively) and then declined (9–12 h and 9–12 h, respectively) during the photoperiod, while during the dark phase (12–24 h), their expressions increased from 12 to 15 h and from 12 to 18 h, respectively, and then declined (15–24 h and 21–24 h, respectively) (Figure 5A,C). For *SaIDH*, its expression levels did not change significantly during the photoperiod but decreased continuously during the dark phase (ANOVA, *p* < 0.05; Figure 5B). In general, the expression levels of both *SaCS* and *Saα-KGDH* showed a trend of increasing and then decreasing at different periods of the circadian cycle. In addition, the cellular ATP content increased gradually during the photoperiod (0–9 h) and decreased gradually in the dark period (12–24 h) (ANOVA, *p* < 0.05; Figure 5D).

### 2.3. The Activity of Energetic Metabolism in Resting Cysts as Reflected in the Expression of Three Targeted Genes, Viability, and ATP Measurements

#### 2.3.1. Expression Levels of *SaCS*, *SaIDH*, and *Saα-KGDH*

**Figure 6:**
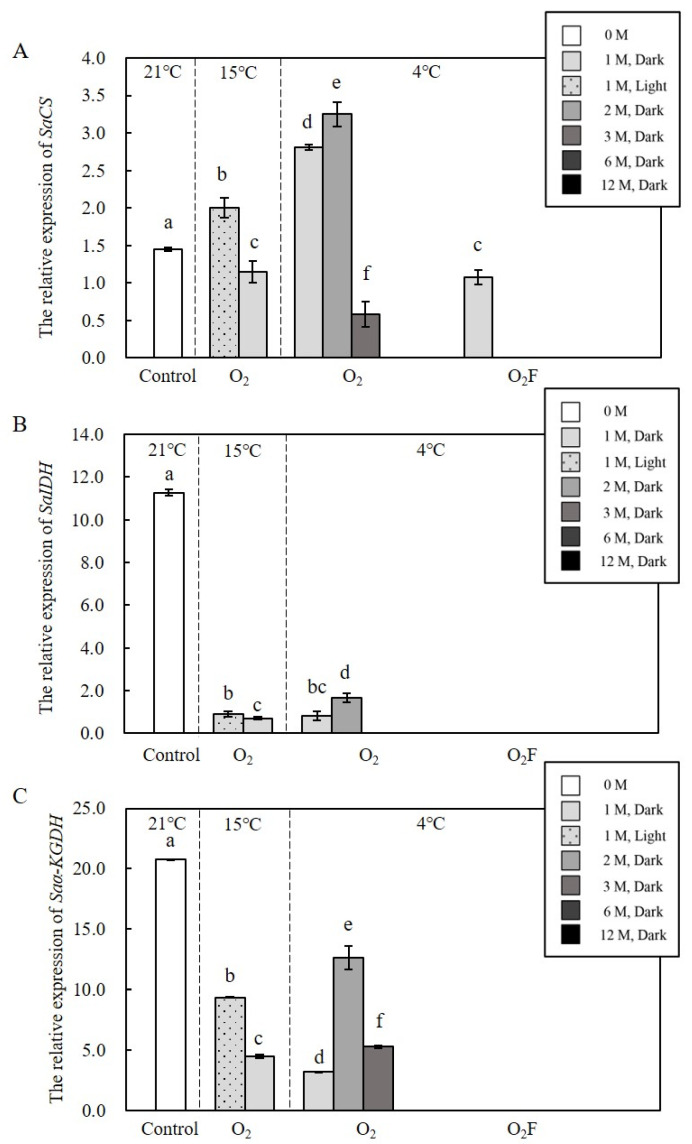
Quantitative expression of *CS*, *IDH*, and *α-KGDH* in resting cysts at different dormancy conditions. (**A**) stood for *CS*; (**B**) stood for *IDH*; (**C**) stood for *α-KGDH*; O_2_ stood for an aerobic environment and O_2_F stood for an anaerobic environment. Different colors represent different lighting and different time processing. Data were presented in graphs as mean ± standard deviation (SD), subjected to one-way analysis of variance (ANOVA) and a subsequent Tukey’s honestly significant difference test, *n* = 13, *p* < 0.05. “a–f” mean that there were differences between the groups they represent, respectively.

Experiments were conducted to investigate the effects of varying temperature, light/dark, oxygen content, and dormancy time on the transcriptional activity of the three targeted genes in resting cysts (Figure 6). At different temperature conditions, the expressions of *SaIDH* and *Saα-KGDH* decreased from the highest at 21 °C to a lower at 15 °C and to the lowest at 4 °C (ANOVA, *p* < 0.05; Figure 6B,C), while the expression of *SaCS* was on the contrary (4 °C > 15 °C > 21 °C; ANOVA, *p* < 0.05; Figure 6A). Under different light conditions (at 15 °C and an aerobic condition), the expressions of three targeted genes were significantly higher under light irradiation than that under darkness (ANOVA, *p* < 0.05; Figure 6A–C). Comparing the effects of aerobic and anaerobic conditions (at 4 °C and darkness), the expressions of three targeted genes in the aerobic condition could be detectable, but the expressions of *SaIDH* and *Saα-KGDH* were below the detection limits in the anaerobic condition (ANOVA, *p* < 0.05; Figure 6A–C). In terms of the responses of gene expression to the prolongation of dormancy time (4 °C, darkness, and the anaerobic), the expressions of all three genes in the second month were higher than that in the first month and then decreased until undetectable (ANOVA, *p* < 0.05; Figure 6A–C).

#### 2.3.2. Cellular ATP Content in All and Live (Viable) Resting Cysts and Its Relation to Neutral Red (NR) Staining-Defined Viability

**Figure 7:**
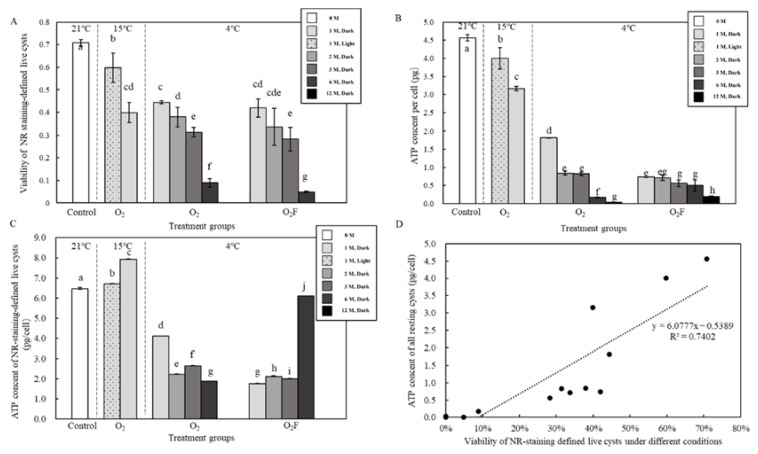
Viability of resting cysts and detection of ATP content in cysts and their correlation. (**A**) The viability of resting cysts measured with NR staining. (**B**) Cellular ATP content of all cysts in sample. (**C**) Cellular ATP content of NR staining-defined live cysts. (**D**) Regression analysis between the cellular ATP content and the NR staining-defined viability. Data were presented in graphs as mean ± standard deviation (SD), subjected to one-way analysis of variance (ANOVA) and a subsequent Tukey’s honestly significant difference test, *n* = 13, *p* < 0.05. “a–j” mean that there were differences between the groups they represent, respectively.

The viability (%) of resting cysts staining defined by NR declined with the decrease in temperature (21 °C to 15 °C, and then to 4 °C), being higher under the light than that under the dark conditions (at 15 °C and aerobic conditions), higher in the aerobic than that in the anaerobic condition, and decreased gradually with the time extension (ANOVA, *p* < 0.05; Figure 7A). The average cellular ATP content also declined gradually with the decrease in temperature (21 °C to 15 °C, then to 4 °C), was higher under the light than under the dark environment, and decreased with increasing dormancy time (ANOVA, *p* < 0.05; Figure 7B). Assuming that only live resting cysts contain ATP, the average cellular ATP content calculated for live resting cysts (ANOVA, *p* < 0.05; Figure 7C) was significantly higher than the ATP content calculated as the ratio of the total ATP content to all resting cysts. The cellular ATP content of the resting cysts changed accordingly and exhibited a manner positively correlated with viability as NR staining defined (R^2^ = 0.7402, *n* = 13, *p* < 0.001; Figure 7D).

## 3. Discussion

In order to maintain cellular viability and germination under suitable conditions, the resting cysts in sediments must undergo efficient energy metabolism. On the one hand, resting cysts also have to consume energy to maintain their viability [42], such as maintaining the basic metabolic capacity to cope with changes in adverse environments. On the other hand, the resting cysts require a minimum level of energy consumption in order to survive as long as possible in the dark, low temperature, and anoxia for even hundreds of years [14,15,16] while retaining sufficient energy to support the germination. Research on the energy metabolism of resting cysts could provide a scientific explanation for the possibility of resting cysts germinating into vegetative cells and returning to the water column. Under appropriate external environmental conditions, only when the resting cysts have reserve energy to support germination can they cause HABs. At present, however, extremely rare studies have been conducted to address the energy metabolism of dinoflagellate cysts, which mainly include the formation and germination processes of resting cysts and pellicle cysts. We previously sequenced the transcriptomes of vegetative cells and resting cysts of *Scrippsiella acuminata* and found that genes involved in the TCA cycle, glycolytic pathway, and glyoxylate cycle were still expressed in resting cysts [18]. A follow-up work also found that the differentially expressed genes related to glycolysis, the TCA cycle, and the ATP level in *S. acuminata* were up-regulated during the formation of pellicle cysts and decreased after the formation of pellicle cysts [19]. Our present study focused on the transcriptional activity of the TCA cycle of resting cysts in response to varying temperature, light/dark, oxygen content, and dormancy time, together with measurements of cellular ATP content and probing the viability of resting cysts, which had gained important insights in the energetic metabolism of resting cysts in the marine sediment.

Compared to the vegetative cells at different growth stages and photoperiods, resting cysts exhibited significantly lower transcription levels of the three TCA cycle pathway genes, indicating much lower energetic levels of dinoflagellate cysts during dormancy. Importantly, the TCA cycle pathway in resting cysts appeared to be largely ceased under an anaerobic environment, as reflected in the undetectable transcriptions of the three key genes and paralleled with a much lower level of the cellular ATP content. Although these findings all seem to be easily predictable based on fundamental cell biology, our work is, to the best of our knowledge, the first demonstrating evidence at levels of gene transcription and measurements of cellular ATP. While the TCA cycle appeared to be inactive in resting cysts under an anaerobic condition, energy metabolic activity, however, was still present in resting cysts as demonstrated in viability and cellular ATP content measurements that decreased gradually to undetectable levels. Therefore, we speculated that in the anaerobic environment of resting cysts, there exist other energetic metabolic pathways providing energy for the viability maintenance of resting cysts. At present, we only select the TCA pathway that produces the most ATP among the energy metabolic pathways for exploration, and we will continue to conduct relevant studies focusing on other energy metabolic pathways in the future.

Under aerobic conditions, resting cysts could still perform the TCA cycle, an observation consistent with the results of Deng et al. [18], and the transcriptional levels of the three targeted genes gradually decreased with the extension of dormancy time. All of the factors examined were found to affect the energy metabolism levels of resting cysts. For temperature, our results obtained that the transcription of the TCA cycle was enhanced by temperature increase (4 °C to 15 °C, then to 21 °C), indicating that the TCA cycle is highly dependent on temperature. Shin et al. [43] obtained the transcriptome of the green alga *Tetraselmis* sp. KCTC12432BP and found that acetyl-CoA oxidation via the TCA cycle was more readily activated at high temperatures compared to low temperatures, suggesting that the expression level of the intracellular TCA cycle may be suppressed under low-temperature conditions. Resting cysts are unable to photosynthesize due to the disappearance of organelles such as chloroplasts [17]. Therefore, resting cysts only carry out energy production processes, whether under light or dark conditions. From Wang [44], light not only induced the transcriptional activities of pyruvate carboxylase and pyruvate dehydrogenase but also increased the activity of key enzymes and intermediates in the TCA cycle, leading to an increased expression level of the TCA cycle. Since the TCA cycle is the hub of multiple energy metabolic pathways in the cell, light conditions can be used to influence the overall energy metabolic activity of the cell by affecting the TCA cycle intermediates. This is confirmed by the ATP content in the resting cysts we obtained, i.e., the ATP content was higher in the light environment than that in the dark environment. Biorhythms can affect the physiological activities in all organisms, such as metabolism, gene expression, and behavioral activities [45,46]. In our study, intracellular ATP levels gradually increased in the light environment and decreased in the dark environment, which corroborates with the conclusion that the TCA cycle may be controlled by a circadian clock system as proposed by Akimoto et al. [47], no matter whether the cell is able to photosynthesize or not. This is an interesting and important observation in the energetic metabolism of dinoflagellate resting cysts, which surely deserves more intensive investigation in the future.

## 4. Materials and Methods

### 4.1. Cultures Maintenance and Resting Cysts Collection

The culture IOCAS-St-1 of *S. acuminata* was originally established from the Yellow Sea, China, and maintained in the Marine Biological Culture Collection Centre, Institute of Oceanology, Chinese Academy of Sciences. Its identity was confirmed via the large subunit (LSU) rDNA sequencing [18]. The culture has been maintained in f/2 (-Si) medium based on seawater with a salinity of 32–33 (Supplementary Materials S2) [48], with the regular addition of penicillin–streptomycin solution at a final concentration of 3% (a mixture of 10,000 I.U. penicillin and 10,000 µg·mL^−1^ streptomycin, Solarbio, Beijing, China). Different batches of the culture were incubated in an incubator (Ningbo Jiangnan Instrument Factory, Ningbo, China) pre-set with an irradiance intensity of 100 µmol photons m^−2^·s^−1^ and a 12:12 h light: dark cycle at 20 ± 1 °C. A large number of resting cysts were formed by adding f/2-Si medium to *S. acuminata* with a one-thousandth dilution of N and P nutrients [49]. After that, resting cysts were harvested at room temperature and rinsed with aseptic seawater, lysozyme, SDS, and sterilized seawater [50].

### 4.2. Samples Treatments

#### 4.2.1. Treatments of Vegetative Cells: Different Growth Stages and Circadian Cycles

Triplicate cultures of *S. acuminata* with an initial density of ~1 × 10^3^ cells·mL^−1^ were grown into 100 mL flasks containing 50 mL medium under the same conditions used for culture maintenance. The culture samples (1 mL) were taken every three days and fixed with Lugol’s solution (final concentration 2%) for counting under an inverted light microscope (IX73, Olympus, Tokyo, Japan) using a Sedgewick Rafter counting chamber. The algal growth curve was plotted by calculating the algal density (Supplementary Materials S3), in which Day 9 was observed to be at the exponential period and Day 15 was the stationary period. For circadian cycles, algal cells cultured at routine maintenance conditions under a 12:12 h light: dark cycle were collected every three hours. A total of 10 samples were yielded, and three biological replicates were performed for each treatment. In order to avoid possible interference with culture volume, at least 30 flasks of cultures were prepared in advance.

#### 4.2.2. Treatments of Resting Cysts: Darkness, Lower Temperatures, and Anoxia

The harvested resting cysts described above were exposed to 13 treatment combinations (Table 1). All of the treatments were conducted independently in triplicate. Dark conditions were accomplished by wrapping test tubes containing cysts in aluminum foil before exposure to different storage temperatures. Low-temperature conditions were conducted in either an incubator or a refrigerator (no light). The anoxia environment was achieved by ventilating the cyst-containing tubes with nitrogen and monitoring the dissolved oxygen (DO) levels of the samples with an oxygen meter (PreSens Microx 4, PreSens, Regensburg, Germany). In addition, the Na-resazurin indicator (0.1% *w*/*v*) was added to the culture medium to ensure anoxia conditions by color change [51].

### 4.3. Full-Length cDNA Cloning and Sequences Analysis of Three Targeted Genes

For cDNA cloning, approximately 5 × 10^4^ vegetative cells were collected by centrifugation for total RNA extraction. The total RNA was extracted in the same procedure as Deng et al. [39] and was subsequently digested using the RNase-Free DNase Set (QIAGEN, Hilden, Germany) according to the manufacturer’s instructions. The RNA quality and concentration were tested with 1% agarose gel electrophoresis and NanoDrop^TM^ 2000 spectrophotometer (Thermo Fisher Scientific, Waltham, MA, USA), respectively.

From the transcriptome database of *S. acuminata* in Deng et al. [18], key gene sequences of *CS*, *IDH*, and *α-KGDH* were selected. The sequence amplification was performed by designing specific primers *CS*-F and *CS*-R, *IDH*-F and *IDH*-R, and *α-KGDH*-F and *α-KGDH*-R, respectively (Supplementary Materials S4). By the RACE PCR, we obtained the full-length cDNAs of *SaCS*, *SaIDH*, and *Saα-KGDH* following the details described by Deng et al. [40]. Primer Premier 5.0 program was used to design specific primers for all genes in this study (see Supplementary Materials S4 for all primers). First-strand cDNA prepared with anchor primers was used as a RACE template. In the 5′-RACE, PCR amplification was performed using DinoSL (specific to dinoflagellates, [52,53] as the forward primer, 5r-*CS*-outer and 5r-*CS*-inner, 5r-*IDH*-outer and 5r-*IDH*-inner, and 5r-*α-KGDH*-outer and 5r-*α-KGDH*-inner as the reverse primers, respectively. In the 3′-RACE, PCR amplification was performed using 3r-*CS*-outer and 3r-*CS*-inner, 3r-*IDH*-outer and 3r-*IDH*-inner, 3r-*α-KGDH*-outer and 3r-*α-KGDH*-inner as the forward primers, and GeneRacer3 (Invitrogen, Karlsruhe, Germany) as the reverse primer, respectively. After amplification, the obtained fragments were purified with a Generay DNA fragment recovery kit (Shanghai, China) and sequenced at the Sangon Biotech Company (Qingdao, China).

The potential protein-coding regions of the identified nucleotide sequences were predicted by the ORF finder [54]. The theoretical molecular weight and isoelectric points of amino acid sequences were predicted by the ProtParam program [55]. Other three species of dinoflagellates containing the sequences of these three genes were downloaded separately from the GenBank database. The corresponding amino acid sequences were compared in multiple sequences using DNAMAN 8.0 software (Lynnon Biosoft, San Ramon, CA, USA) (Supplementary Materials S1).

### 4.4. Transcriptional Profiles of SaCS, SaIDH and Saα-KGDH with Real-Time PCR (RT-qPCR) Detection

The expression levels of *SaCS*, *SaIDH*, and *Saα-KGDH* in *S. acuminata* exposed to different treatments were determined using RT-qPCR. All RT-qPCRs were performed in 96-well plates on Light Cycler^®^ 480 System (Roche, Pleasanton, CA, USA) with TB Green^®^ Premix Ex Taq^TM^ II (Takara, Otsu, Shiga, Japan). The single-strand cDNAs were prepared with random primers using the PrimeScript RT Kit (Takara, Japan). Specific primers *qCS*, *qIDH*, and *qα-KGDH*, were designed based on the full-length sequences of *SaCS*, *SaIDH*, and *Saα-KGDH* (see Supplementary Materials S4 for the sequences of these primers). The ubiquitin-conjugating enzyme (UBC) gene was selected as the internal control [18]. The single peak melting curve analysis was performed to demonstrate the amplification specificity. Three technical replicates were prepared for all reactions. The protocol used was as follows: 30 s at 95 °C, followed by 40 cycles of 5 s at 95 °C, 30 s at 50 °C, and 30 s at 72°C. The 2^−∆∆Ct^ relative quantification method was used to analyze the difference in gene expression. Statistical analyses were conducted with the software SPSS 22, and the significance level was set at *p* = 0.05. One-way analysis of variance (ANOVA) was performed for all experimental data.

### 4.5. Viability Measurements of Resting Cysts via NR Staining

Neutral red (NR; 3-amino-7-dimethylamino-2-methylphenazine hydro-chloride) has been used as a vital stain to measure a cell’s or individual’s viability [56,57,58,59]. Recently, we also successfully applied NR-staining to detect the viability of resting cysts of *S. acuminata* [42] and *Gymnodinium catenatum* [60]. The assay used in the present work followed the method of Li et al. [42]. Briefly, resting cysts were stained with NR (Solarbio Science and Technology, Beijing, China) at a final concentration of 0.33% and a pH adjusted to ~7.0 for 24 h at room temperature. The viability of the resting cysts was defined as the percent of cells (cysts) that could be stained by NR (look brightly red under bright field of LM). It can be calculated by counting both NR-stained and unstained cysts using a Sedgewick Rafter counting chamber (1.0 mL of the sample) and an inverted light microscope (IX73, Olympus, Tokyo, Japan). Three parallel samples were prepared for each treatment (group), and at least 1 × 10^3^ cysts were examined for each sample.

### 4.6. Measurements of Cellular ATP Content in Vegetative Cells and Resting Cysts

The QuenchGone^TM^ Aqueous Test Kit (LuminUltra, Fredericton, NB, Canada) combined with a spectrophotometer (LuminUltra, Fredericton, NB, Canada) was used to quantify the cellular ATP content, which has been successfully applied to cyanobacteria and plankton in ballast water [61,62]. The detection procedure followed that of Li et al. [42]. Firstly, all of the cyst samples were crushed with a TGrinder High-Speed Tissue grinder (OSE-Y30, Tiangen Biotech, Beijing, China) at room temperature for 90 s; secondly, the detection value RLU_ATP1_ was recorded by calibrating with luciferase and standards according to QuenchGone^TM^ Aqueous Test Kit (LuminUltra, Fredericton, NB, Canada) and PhotonMaster Luminometer (EQP-PMT) (LuminUltra, Fredericton, NB, Canada); after that, the RLU_cATP_ values of each sample were detected; finally, the cellular ATP content was calculated using the formula: cATP = RLU_cATP_/RLU_ATP1_ × 10^4^/N_sample_, where cATP indicates cellular ATP content; RLU indicates relative light units, i.e., Lux/OD600; and N_sample_ indicates the number of cysts.

## 5. Conclusions

Combining the above-described results and the parallel changing trends of the transcription of genes, NR staining-defined cyst viability, and cellular ATP content, we can conclude that the TCA cycle in resting cysts is still an essential pathway for energy catabolism under aerobic conditions, and its transcriptional expression is significantly enhanced at higher temperatures, light irradiation, and the early stage of dormancy. It is worth mentioning that intracellular ATP levels are more active in light than dark environments, regardless of the presence of intracellular photosynthesis. In addition, under anaerobic conditions, the TCA cycle pathway ceased to be expressed in resting cysts. Our results together have laid out a cornerstone for reaching a comprehensive understanding of the molecular mechanisms of energy metabolism and viability maintenance in resting cysts that are buried in marine sediments experiencing different time lengths and environmental conditions.

## Figures and Tables

**Table 1:** Dormancy conditions of resting cysts.

Treatments	Temperature (°C)	Oxygen	Light	Dormancy Time (Month)
Control	21	Yes	Yes	0
No.1	15	Yes	Yes	1
No.2	15	Yes	No	1
No.3	4	Yes	No	1
No.4	4	Yes	No	2
No.5	4	Yes	No	3
No.6	4	Yes	No	6
No.7	4	Yes	No	12
No.8	4	No	No	1
No.9	4	No	No	2
No.10	4	No	No	3
No.11	4	No	No	6
No.12	4	No	No	12

## Data Availability

The data are contained within the article and supplementary material.

## Supplementary Materials

The supporting information can be downloaded at: https://www.mdpi.com/article/10.3390/ijms232315033/s1.
