# Supplementary material for: Characterizing the Status of Energetic Metabolism of Dinoflagellate Resting Cysts under Mock Conditions of Marine Sediments via Physiological and Transcriptional Measurements
Source: Int J Mol Sci. 2022 Nov 30;23(23):15033. doi: 10.3390/ijms232315033 (PMC9739985; doi:10.3390/ijms232315033)
Supplement: Supplementary file 1 [file ijms-23-15033-s001.zip › Supplementary Materials S1.pdf]

Supplementary materials S1. Characterizing the status of energetic metabolism of dinoflagellate resting cysts under mock conditions of marine sediments via physiological and transcriptomic measurements

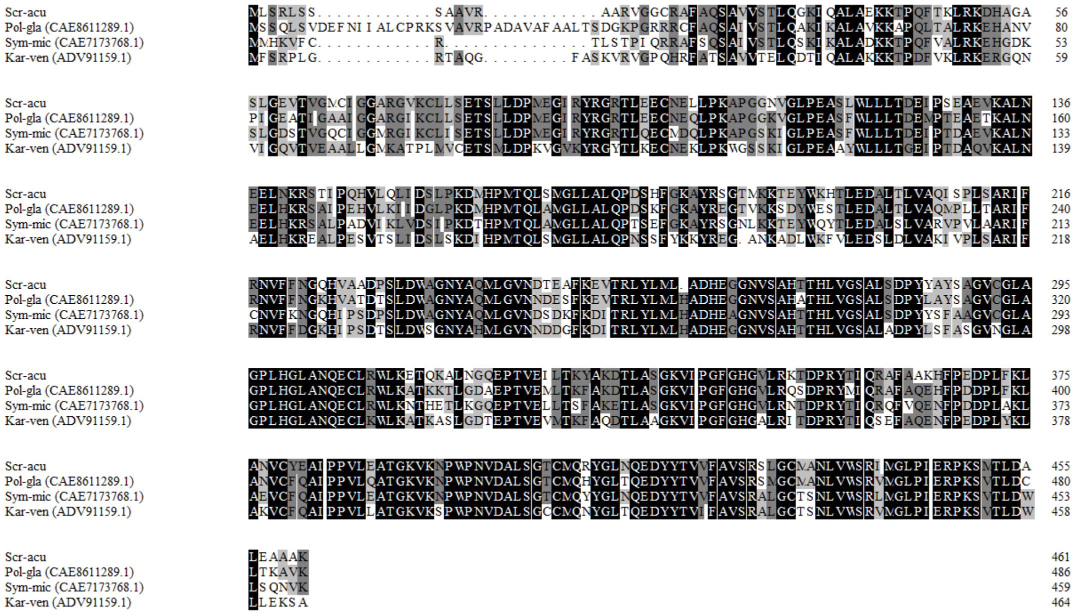

Figure S1-1 Multiple sequence alignment of *SaCS* amino acid sequences with other known dinoflagellate species.The shaded areas indicated similarities. Scr-acu=*S. acunimata*, Pol-gla=*Polarella glacialis*, Sym-mic= *S. microadriaticum*, Kar-ven=*K. veneficum*.

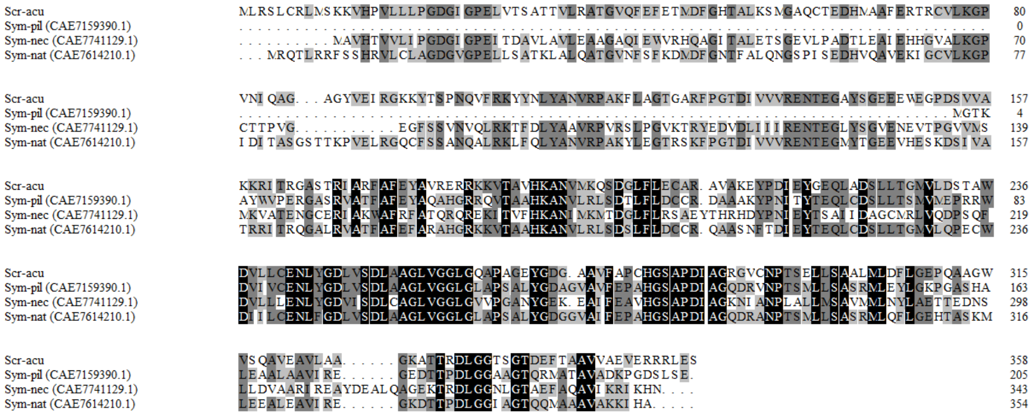

Figure S1-2 Multiple sequence alignment of *SaIDH* amino acid sequences with other known dinoflagellate species. The shaded areas indicate similarities. Scr-acu= *S. acuminata*, Sym-pil= *S. pilosum*, Sym-nat= *S. natans*, Sym-nec= *S. necroappetens*.

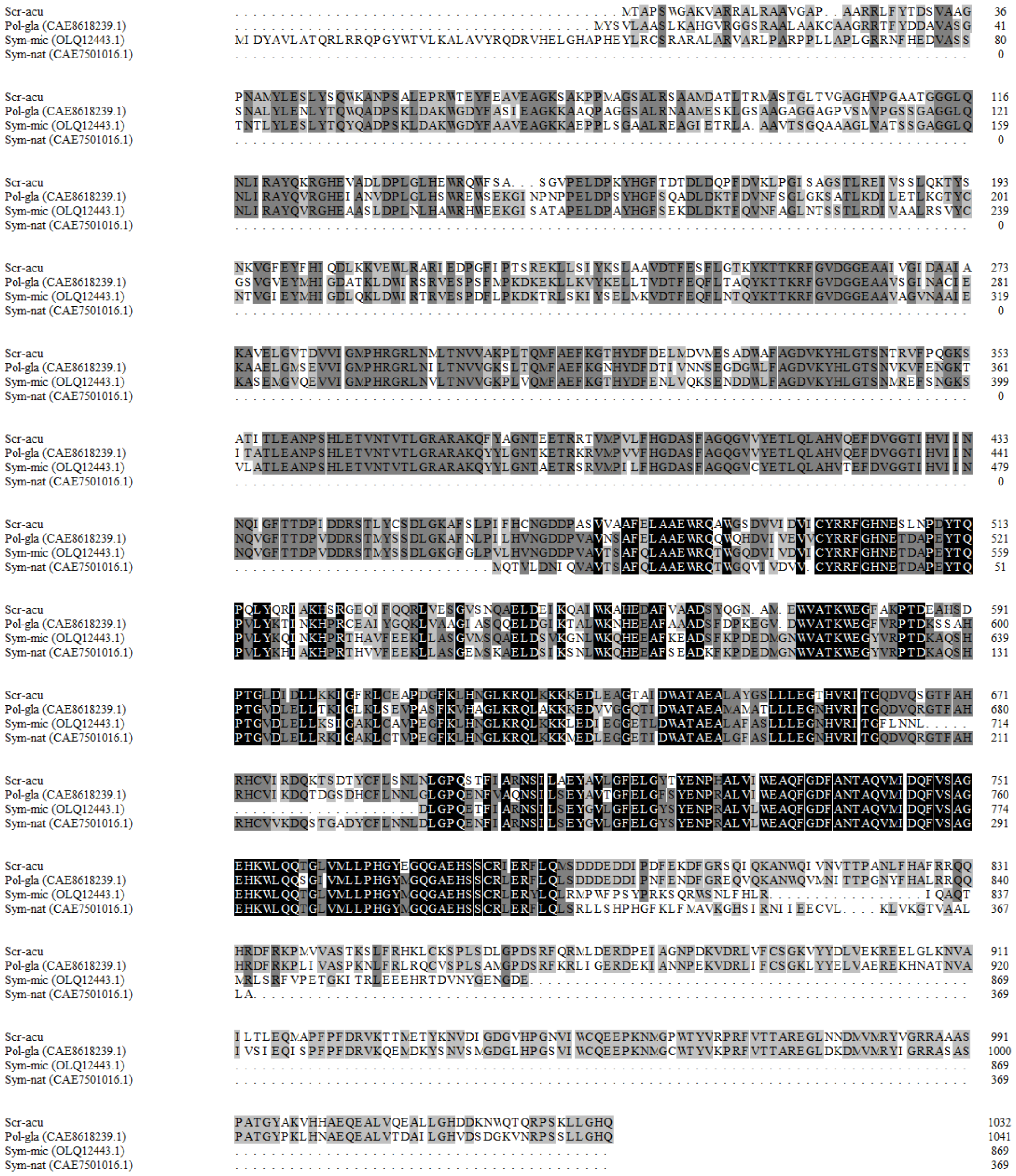

Figure S1-3 Multiple sequence alignment of *Saα-KGDH* amino acid sequences with other known dinoflagellate species. The shaded areas indicate similarities. Scr-acu= *S. acuminata*, Pol-gla= *P. glacialis*, Sym-mic= *S. microadriaticum*, Sym-nat= *S. natans*.
