# Supplementary material for: Characterizing the Status of Energetic Metabolism of Dinoflagellate Resting Cysts under Mock Conditions of Marine Sediments via Physiological and Transcriptional Measurements
Source: Int J Mol Sci. 2022 Nov 30;23(23):15033. doi: 10.3390/ijms232315033 (PMC9739985; doi:10.3390/ijms232315033)
Supplement: Supplementary file 1 [file ijms-23-15033-s001.zip › Supplementary Materials S2.pdf]

Supplementary materials S2. Characterizing the status of energetic metabolism of dinoflagellate resting cysts under mock conditions of marine sediments via physiological and transcriptomic measurements

Table S1-1. Information of nutrient concentrations (Guillard, 1975)

Component	Molar Concentration in Final Medium
$NaNO_3$	8.82×10^{-4} M
$NaH_2PO_4 \cdot H_2O$	3.62×10^{-5} M
$FeCl_3 \cdot 6H_2O$	1.17×10^{-5} M
$Na_2EDTA \cdot 2H_2O$	1.17 × 10-5 M
CuSO4 $5H_2O$	3.93×10^{-8} M
$Na_2MoO_4 \cdot 2H_2O$	2.60×10^{-8} M
$ZnSO_4 \cdot 7H_2O$	7.65×10^{-8} M
$CoCl_2 \cdot 6H_2O$	4.20×10^{-8} M
$MnCl_2 \cdot 4H_2O$	9.10×10^{-7} M
thiamine HCl (vit. B_1)	2.96×10^{-7} M
biotin (vit. H)	2.05×10^{-9} M
cyanocobalamin (vit. B_{12})	3.69×10^{-10} M

Reference:

Guillard, R. R. Culture of phytoplankton for feeding marine invertebrates. *Culture of marine invertebrate animals* **1975**, pp 29-60.
