# Supplementary material for: Characterizing the Status of Energetic Metabolism of Dinoflagellate Resting Cysts under Mock Conditions of Marine Sediments via Physiological and Transcriptional Measurements
Source: Int J Mol Sci. 2022 Nov 30;23(23):15033. doi: 10.3390/ijms232315033 (PMC9739985; doi:10.3390/ijms232315033)
Supplement: Supplementary file 1 [file ijms-23-15033-s001.zip › Supplementary Materials S3.pdf]

Supplementary materials S3. Characterizing the status of energetic metabolism of dinoflagellate resting cysts under mock conditions of marine sediments via physiological and transcriptional measurements

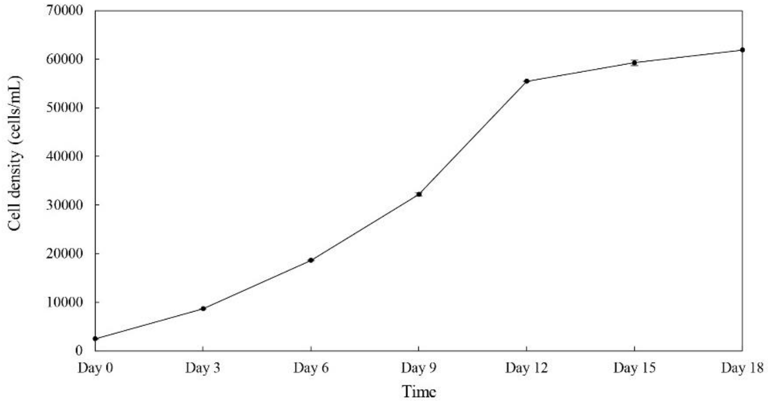

Figure S2. The growth curve of *Scrippsiella acuminata* (strain IOCAS-St-1). The cell densities were counted using a Sedgewick Rafter counting chamber (1.0 mL of sample) under an inverted light microscope (IX73, Olympus, Tokyo, Japan). The day of inoculation was recorded as Day 0. Values are mean ± standard deviation, Error Bars = SD, *n*=3.
