# Supplementary material for: Characterizing the Status of Energetic Metabolism of Dinoflagellate Resting Cysts under Mock Conditions of Marine Sediments via Physiological and Transcriptional Measurements
Source: Int J Mol Sci. 2022 Nov 30;23(23):15033. doi: 10.3390/ijms232315033 (PMC9739985; doi:10.3390/ijms232315033)
Supplement: Supplementary file 1 [file ijms-23-15033-s001.zip › Supplementary Materials S4.pdf]

Supplementary materials S4. Characterizing the status of energetic metabolism of dinoflagellate resting cysts under mock conditions of marine sediments via physiological and transcriptomic measurements

Table S2. Information of primers used in the present study

Primer Name	Nucleotide Sequences (5'-3')	Amplicon Length	Description
CS-F	CTTGTGGCTCTTGTTGACCG	519 bp	*SaCS* fragment amplification
CS-R	GCGGAGTAGGCGTAGTAGGG		
5r-*CS*-outer	CGAAATGGCTGTCCGGCTGC	499 bp	*SaCS* 5' RACE-PCR
5r-*CS*-inner	TTGACCTCAGCCTCGCTCGGGA		
3r-*CS*-outer	TGGGCCGGGAACTACGCGC	790 bp	*SaCS 3' RACE-PCR*
3r-*CS*-inner	CGACACCGAGGCGTTCAAGGAG		
IDH-F	AACCTCTACGCAAATGTTCGG	367 bp	*SaIDH* fragment amplification
IDH-R	GCACCATCCCTGTGAGCAAC		
5r-*IDH*-outer	GAGGCTCCCCGTGTGATGCGTT	537 bp	*SaIDH* 5' RACE-PCR
5r-*IDH*-inner	CCTCCCACTCCTCCTCGCCTGA		
3r-*IDH*-outer	AGGCGAGGAGGAGTGGGAGGGG	791 bp	*SaIDH* 3' RACE-PCR
3r-*IDH*-inner	GGAGCGGCGAAAGAAAGTGACC		
α-KGDH-F	CGGGTTCACCGATACAGA	1429 bp	*Saα-KGDH* fragment amplification
α-KGDH-R	GCCTCCAAATCCTCCTTTT		
5r-*α-KGDH*-outer	GATGATTACGTGGATCGTGCCCCC	618 bp	*Saα-KGDH* 5' RACE-PCR
5r-*α-KGDH*-inner	CCAGCACTTATCCCAGGGAGCTTTACGT		
3r-*α-KGDH*-outer	CGAAGCACTCGCGGGGAGAACAGA	1650 bp	*Saα-KGDH* 3' RACE-PCR
3r-*α-KGDH*-inner	TCAGGGGAATGCGATGGAGTGGGT		
q*CS*-F	AGATGCTCGGGGTGAAC	151 bp	qPCR for *SaCS*
q*CS*-R	CGGAGTAGGCGTAGTAGGG		
q*IDH*-F	ACTACAACCTCTACGCAAATG	127 bp	qPCR for

q*IDH*-R	CACTCCTCCTCGCCTGA		*SaIDH*
q*α-KGDH*-F	GATGAAGCACATTCTGACCC	111 bp	qPCR for *Saα-KGDH*
q*α-KGDH*-R	CCGTTTGAGGCCGTTG		
q*UBC*-F	GTCTTGACCTACTACGTGGAGC	172 bp	qPCR for *UBC*
q*UBC*-R	CGGGCGTTGTACTGATGG		
